# Hepatocellular carcinoma-targeted drug discovery through image-based phenotypic screening in co-cultures of HCC cells with hepatocytes

**DOI:** 10.1186/s12885-016-2816-x

**Published:** 2016-10-18

**Authors:** Jae-Woo Jang, Yeonhwa Song, Kang Mo Kim, Jin-Sun Kim, Eun Kyung Choi, Joon Kim, Haengran Seo

**Affiliations:** 1 Cancer Biology Research Laboratory, Institut Pasteur Korea, 16, Daewangpangyo-ro 712 beon-gil, Bundang-gu, Seongnam-si, Gyeonggi-do 13488 Korea; 2 Laboratory of Biochemistry, Division of Life Sciences, Korea University, 145, Anam-ro, Seongbuk-gu, Seoul, 02841 Korea; 3 Division of Gastroenterology and Hepatology, ASAN Medical center, Olympic-ro 43-gil, Songpagu, Seoul 05505 Korea; 4 Division of Radiation Oncology, ASAN Medical center, Olympic-ro 43-gil, Songpa-gu, Seoul 05505 Korea

**Keywords:** Hepatocellular carcinoma, Phenotypic screening, Co-cultures, Pyrimethamine, 3D culture systems

## Abstract

**Background:**

Hepatocellular carcinoma (HCC) is one of the most common malignant cancers worldwide and is associated with substantial mortality. Because HCCs have strong resistance to conventional chemotherapeutic agents, novel therapeutic strategies are needed to improve survival in HCC patients.

**Methods:**

Here, we developed a fluorescence image-based phenotypic screening system in vitro to identify HCC-specific drugs in co-cultures of HCC cells with hepatocytes. To this end, we identified two distinctive markers of HCC, CHALV1 and AFP, which are highly expressed in HCC cell lines and liver cancer patient-derived materials. We applied these markers to an HCC-specific drug screening system.

**Results:**

Through pilot screening, we identified three anti-folate compounds that had HCC-specific cytotoxicity. Among them, pyrimethamine exhibited the greatest HCC-specific cytotoxicity. Interestingly, pyrimethamine significantly increased the size and number of lysosomes and subsequently induced the release of cathepsin B from the lysosome to the cytosol, which triggered caspase-3-dependent apoptosis in Huh7 (HCC) but not Fa2N-4 cells (immortalized hepatocytes). Importantly, Fa2N-4 cells had strong resistance to pyrimethamine relative to Huh7 cells in 2D and 3D culture systems.

**Conclusion:**

These results demonstrate that this in vitro image-based phenotypic screening platform has the potential to be widely adopted in drug discovery research, since we promptly estimated anticancer activity and hepatotoxicity and elucidated functional roles of pyrimethamine during the apoptosis process in HCC.

**Electronic supplementary material:**

The online version of this article (doi:10.1186/s12885-016-2816-x) contains supplementary material, which is available to authorized users.

## Background

Hepatocellular carcinoma (HCC) is the seventh most common malignant cancer and the third leading cause of cancer-related deaths in the world [1–3]. Over the past decade, the advancements in medical device development, surgical techniques, radiology, liver transplantation, and other therapies have resulted in considerable improvements in HCC treatment [1, 4]. However, the numbers of incident cases and liver cancer deaths have still increased because most HCCs are detected at an advanced stage in patients with underlying liver dysfunction, making it a highly lethal cancer. Moreover, most HCCs are resistant to conventional chemotherapeutic agents, and patients with HCC usually have poor tolerance to systemic chemotherapy because of underlying liver dysfunction. In these situations, liver-targeted drugs with fewer side effects and high efficacy are a desired but unmet need for the treatment of liver cancer.

In this study, we used a fluorescence image-based phenotypic screening approach to specifically target HCC mixed with normal hepatocytes, because heterologous cell types within tumors can actively influence the therapeutic response and shape resistance. Specifically, we aimed to develop liver cancer-specific drugs that could induce cell death in HCC cells, while minimizing the damage to normal hepatocytes, in a mixed cell culture system containing hepatocytes and HCC cells. To distinguish between HCC cells and hepatocytes in this mixed cell culture system, we identified two well established markers of HCC, CHALV1 and AFP, which are highly expressed in HCC cell lines and liver patient samples [5, 6]. Given the heterogeneity of tumor tissues, our approach is expected to be positively utilized for drug discovery.

Anti-folate drugs are used in cancer chemotherapy and act through the inhibition of dihydrofolate reductase (DHFR). Since the inhibition of DHFR blocks nucleotide biosynthesis, anti-folate drugs reduce the proliferation of cancer cells [7]. In particular, pyrimethamine (2,4-diamino-5-p-chlorophenyl-6-ethyl-pyrimidine), a folic acid antagonist, is used to treat protozoal infections. It is also used as an antimalarial drug and a treatment for *Toxoplasma gondii* infections in immunocompromised patients [8–10]. Recent findings showed that pyrimethamine effectively induces apoptosis in pituitary adenoma cells, peripheral blood lymphocytes, and melanoma cells [11–13]. Although pyrimethamine has feasibility as an anticancer drug, its anticancer effects and functional roles have not been established in HCC. Here, we identified a hitherto unknown mechanism of pyrimethamine-induced apoptosis in HCC cells using fluorescence image-based phenotypic analysis. In order to assess pyrimethamine-induced phenotypic changes and cytotoxic effects in HCC, we applied various cell-based assay models in vitro to the High Content Screening system. We also applied a hepatocellular 3D culture method to this system, which is the appropriate culture model to maintain liver-specific functions and to validate drug efficiency.

Based on these applications, we established an image-based phenotypic screening platform for HCC-specific drug discovery and the functional study of interesting compounds. Additionally, we found that pyrimethamine induced HCC death via lysosome modification and activation of cathepsin B.

## Methods

### Cell culture and labeling

Fa2N-4 cells (an immortalized normal hepatocyte cell line) were purchased from Xenotech (Lenexa, KS, USA), and Huh7, Hep3B, PLC/PRF/5, SNU475 and SNU449 (human hepatocellular carcinoma cell line) were obtained from the Korean Cell Line Bank (KCLB). Huh7.5 [14] was kindly provided by Charles M. Rice (Rockefeller University, New York, USA), and Huh6 [15] was kindly provided by Dr. Ralf Bartenschlager (University of Heidelberg, Germany). Cells were maintained at 37 °C with 95 % humidity and 5 % CO_2_. After cell attachment (3–6 h), serum-containing plating medium (XenoTech, Lenexa, KS, USA) was replaced with MFE serum free supporting Fa2N-4 cells (SF) medium (XenoTech) which are nutrient rich medium for maintaining Fa2N-4 cells in culture. This is a serum free medium. Huh7 cells (a human HCC cell line) were cultured in Dulbecco’s modified Eagle’s medium (DMEM; Gibco, Gaithersburg, MD, USA) supplemented with heat-inactivated 10 % fetal bovine serum (FBS; Gibco) and antibiotics (Gibco) at 37 °C in a humidified incubator under 5 % CO_2_. For the 3D culture, 8 μl of Matrigel (BD Biosciences, San Jose, CA, USA) was pipetted directly onto the surface and carefully spread to avoid bubbles in 384 well culture plates (Greiner Bio-One, Monroe, NC, USA), then incubated at 37 °C until the Matrigel solidified. Trypsinized single cells from a monolayer were centrifuged at 1,000 rpm, resuspended in 30 ml of supporting culture medium, and plated onto the Matrigel-coated plates at a density of 2 × 10^3^ cells/well. Cells were incubated for 30 min at 37 °C to settle onto the Matrigel, then 10 % Matrigel-Medium was slowly added to each well. After maintaining for 5 days, the Matrigel-Medium was replaced every 2 days.

To distinguish between the Fa2N-4 and Huh7 cells in the mixed culture system, Fa2N-4 cells were labeled with CellLight® Nucleus-GFP (Thermo Fisher Scientific, Marietta, OH, USA). Fa2N-4 cells were infected with BacMam expression vectors encoding fusions of GFP with the SV40 nuclear localization sequence at 30 particles per cell, according to the manufacturer’s instructions.

### Primary cell culture

Isolated liver cancer tissues were cut into 3 mm^3^ pieces and washed with 4 °C Hank’s balanced salt solution (Lonza, Walkersville, MD, USA) supplemented with 1× antibiotic antimycotic solution (Sigma, St Louis, MO, USA) and 1× penicillin streptomycin (Lonza) in a 100-mm petri dish, then moved to a 15-ml conical tube. Cells were washed three times with bovine serum solution (BS solution) consisting of Dulbecco’s modified Eagle’s medium: nutrient mixture F-12 (DMEM/F12; Gibco) supplemented with 1× antibiotic antimycotic solution (Sigma), 1× penicillin streptomycin (Lonza), and 10 % bovine serum (Gibco). Then, the cells were resuspended with 10 ml of BS solution and incubated at 4 °C for 16 h. After removing the BS solution and washing with fresh BS solution, tissues were incubated with 2 ml of 2× collagenase II (BD Biosciences) at 37 °C in a shaking chamber for 90 min. After incubation, 10 ml of BS solution was added and the sample was centrifuged at 600 rpm for 2 min. This washing step was performed several times until the supernatant became clear. The pellet was resuspended in hepatocyte basal medium (HBM; Lonza) containing 1× antibiotic antimycotic solution (Sigma), hepatocyte culture medium growth factor solution (Lonza), 10 % FBS (Gibco), and 5 μg/ml of hepatocyte growth factor (HGF; R&D Systems, Minneapolis, MN, USA), and the cells were plated in a collagen type I-coated T-25 flask (BD Biosciences). After incubation for 24 h at 37 °C in an incubator, the cells were washed with phosphate buffered saline (PBS; Lonza) containing 1× antibiotic antimycotic (Sigma) and were replaced with fresh HBM media containing supplements (Lonza).

### Reagents and antibodies

Tetramethylrhodamine, methyl ester (TMRM), Hoechst 33342, LysoTracker® Red DND-99, LysoTracker® Green DND-26, 5-(and-6)-chloromethyl-2′,7’-dichlorodihydrofluorescein diacetate, acetyl ester (CM-H_2_DCFDA), CellLight® Nucleus-GFP, Alexa Fluor® 633 phalloidin, goat anti-mouse Alexa Fluor® 633, goat anti-rabbit Alexa Fluor® 633, goat anti-mouse Alexa Fluor® 488, and goat anti-rabbit Alexa Fluor® 488 were purchased from Molecular Probes (Thermo Fisher Scientific). Resazurin, pyrimethamine, methotrexate, aminopterin, and most of the drugs were purchased from Sigma. The rabbit polyclonal anti-AFP (Dako, Denmark A/S, Denmark), mouse monoclonal anti-CHALV1 (Abcam, CSP, Cambridge, England), rabbit polyclonal anti-cleaved caspase-3 (Cell Signaling Technology, Danvers, MA, USA), mouse monoclonal anti-phospho-histone H2AX (γ-H2AX) (Millipore, Bedford, MA, USA), and rabbit polyclonal anti-cathepsin B (EMD/Calbiochem, San Diego, CA, USA) antibodies were purchased from each of the indicated companies.

### Resazurin assay

Cells were seeded at 5 × 10^4^ cells/well into 96 well microtiter plates (BD Biosciences) and cultured. On the next day, cells were treated with hepatotoxic or safety drugs. After 3 days, cells were supplemented with 44 μM resazurin in the medium. Eight hr later, resazurin reduction was measured colorimetrically (570/600 nm) using a Victor3 (Perkin Elmer, Waltham, MA, USA) plate reader.

### High Content Screening (HCS) System

After being treated with the indicated concentrations of various drugs for 24 h, the cells were washed with PBS and stained by fluorescent probes, including TMRM, Hoechst 33342, and CM-H_2_DCFDA. The cells and probes were incubated together for the first 30 min. Automated live-cell multispectral image acquisition was performed on the Operetta® High Content Screening System using a 20× objective (Perkin Elmer). The fluorescence images were captured according to the following optimal excitation and emission wavelengths of each probe:

- 485 ± 20 and 515 ± 10 nm for CM-H_2_DCFDA (ROS)
- 532 ± 4 and 600 ± 12.5 nm for TMRM (MMP, mitochondrial membrane potential)
- 405 ± 25 and 455 ± 10 nm for Hoechst 33342 (nuclei)

To capture enough cells (> 100) for the analysis, four image fields were collected from each well, starting at the center. All of the image analysis was performed using Operetta and Harmony 3.5.1 software (Perkin Elmer). A series of measurements from the nuclei, lipids, ROS, and TMRM channel images were obtained for each drug.

### Confocal immunofluorescence analysis

For immunofluorescence analysis, cells were fixed with 4 % paraformaldehyde (PFA, Sigma), permeabilized with 0.1 % Triton X-100 (Sigma) in PBS, and then washed three times with PBS. Cells were then incubated with anti-cleaved caspase-3 (Asp175) or anti-cathepsin B in PBS with 10 % normal goat serum (Vector Laboratories, Burlingame, CA, USA) for 12 h at 4 °C in a humidified chamber. Excess antibody was removed by washing three times with PBS. Cells were then incubated with fluorescein-conjugated secondary antibody (Molecular Probes) at a 1:200 dilution in PBS for 1 h at room temperature. Actin was visualized by Alexa Fluor® 633 phalloidin and nuclei were co-stained with Hoechst 33242 in 3D spheroids. After washing five times with PBS, automated cell multispectral image acquisition was performed on the Operetta® High Content Screening System at a 2 μm interval using a 20× objective (Perkin Elmer). The numbers of stacks varied according to the marker visualized, as follows:

- 405 ± 25 and 455 ± 10 nm for Hoechst 33342
- 488 ± 20 and 515 ± 10 nm for cleaved caspase-3 and cathepsin B (apoptosis markers)
- 532 ± 20 and 560 ± 10 nm for LysoTracker® Red DND-99 (lysosome)
- 655 ± 15 and 730 ± 25 nm for Alexa Fluor® 633 phalloidin (F-actin)

### Immunohistochemistry

Various cancer tissues with surrounding normal tissues were arrayed from formal formalin-fixed and paraffin-embedded tissues on an AccuMax array (Petagen Inc., Seoul, Korea), and these arrayed slides were used for the immunostaining of CHALV1 and AFP. Deparaffinization and rehydration were performed using xylene and ethanol (Sigma), and pretreated slides were incubated in 3 % H_2_O_2_ (Sigma) for 13 min to remove endogenous peroxidase activity. The tissue was reacted with primary anti-CHALV1 and AFP for 16 h at 4 °C and washed with PBS for 10 min, and the sections were incubated for 2 h at room temperature with goat anti-rabbit Alexa Fluor® 633 and goat anti-mouse Alexa Fluor® 488 secondary antibodies. After washing three times with PBS, coverslips were mounted onto microscope slides using ProLong antifade mounting reagent (Molecular Probes). The slides were analyzed using the Operetta® High Content Screening System (Perkin Elmer).

### Polyacrylamide gel electrophoresis (PAGE) and Western blot analysis

For PAGE and Western blot analysis, cells were solubilized with lysis buffer (Sigma), boiled for 5 min, and an equal amount of protein per well (50 μg) was analyzed by 10 % SDS-PAGE. After electrophoresis, proteins were transferred onto a polyvinylidene difluoride (PVDF) membrane (GE Healthcare Life Sciences, Piscataway, USA) and processed for immunoblotting. Blots were further incubated with horseradish peroxidase-conjugated secondary antibody (Santa Cruz Biotechnology, Dallas, TX, USA) diluted at 1:5,000, and specific bands were visualized using a chemiluminescent substrate (ECL; Thermo Fisher Scientific). Autoradiographs were recorded onto X-Omat AR films (Eastman Kodak Co., Rochester, NY, USA).

### Screening procedure

#### Step 1: Pilot screening

1.5×10^3^ cells/well of Fa2N-4 and 0.8×10^3^ cells/well of Huh7 were mixed and plated in 384 well plate. After 16 h, 10 μM of 43 compounds (see Additional file 1: Figure S5) were treated to each well in duplicate. After 3 days incubation, cells were fixed with 4 % PFA and permeabilized with 0.1 % Triton X-100. The cells were reacted with primary anti-CHALV1 and AFP for 16 h at 4 °C and washed with PBS for 10 min, and were incubated for 2 h at room temperature with goat anti-rabbit Alexa Fluor® 633 and goat anti-mouse Alexa Fluor® 488 secondary antibodies. After three times washing with PBS, cells were stained with Hoechst 33342 for nucleus. Cell images were obtained by Operetta® High Content Screening System and analyzed by Harmony 3.5.1® high content imaging and analysis software (Perkin Elmer).

Sorafenib was treated as positive control and Z’ score was calculated by Harmony High-Content Imaging and Analysis Software using positive and negative (DMSO treatment) control. The compounds that > 50 % Huh7 and < 20 % Fa2N-4 inhibition were selected.

#### Step 2: Hit confirmation

The selected compounds were confirmed by resazurin assay after individual treatment to Huh7 and Fa2N-4 cells at 10 point concentrations (DMSO control, 1 pM to 100 μM). For statistical analysis of IC_50_ values, GraphPad Prism was used after resazurin assays. The values of IC_50_ were calculated by nonlinear regression model with a sigmoidal dose response. Experiments were performed in triplicate. Statistical analysis was performed by Student’s *t* test (*p* < 0.05).

#### Step 3: Secondary assay

The selected compounds were re-confirmed by Step 1 screening system at 10 point concentrations.

#### Step 4: MoA (Mode of action) study

The mechanisms of anticancer effect on selected compounds were studied using HCS system and Western blot analysis.

### Small interfering RNA (siRNA) transfection

Fa2N-4 and Huh7 cells were transfected with On-TARGET *plus* Human cathepsinB siRNA (siCTSB; Dharmacon, Lafayette, CO, USA). The sequences of siCTSB were as follows: siCTSB #1, 5’-GGAUCACUGUGGAAUCGAA-3’, siCTSB #2, 5’-GCACAACUUCUACAACGUG-3’, siCTSB #3, 5’-GAGGCUAUGUGGUACCUUC-3’, siCTSB #4, 5’-GCACCGAUCAGUACUGGGA-3’. Cells were then transfected with these siRNAs for 48 h using Lipofectamine® RNAiMAX (Invitrogen).

### Caspase 3/7 activity assay

Caspase-Glo® 3/7 Assay Systems (Promega, Madison, WI, USA) was utilized to detect DEVDase (caspase-3/7) activity. Briefly, cathepsin B knock-downed Huh7 and Fa2N-4 cells were treated with pyrimethamine, after which they were lysed in the lysis buffer provided in the kit. The protein content was then determined, after which the lysates were incubated with Ac-DEVD-pNA for 4 h at room temperature at 405 nm.

### Statistical analysis

All experiments were performed at least three times. The results are expressed as the mean ± standard deviation (SD). Statistical analysis was performed using the Student’s *t*-test.

## Results

### CHALV1 and AFP are appropriate markers to distinguish between HCCs and hepatocytes

**Fig. 1:**
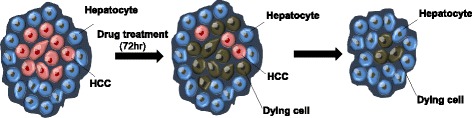
Schematic illustration of a mixed culture system for the High Content Screening of HCC-specific targeting drugs. A mixed culture system, which is composed of HCC cells and normal hepatocytes, can be used to screen for HCC-specific targeting drugs

We aimed to develop liver cancer-specific compounds that induce cell death in HCC cells, while minimizing the damage to hepatocytes, by creating a mixed cell culture system. For HCC-specific drug screening, the co-culture system was composed of HCC cells and hepatocytes (Fig. 1). Because two different populations were present in the co-culture system, we first sought to find HCC-specific markers to distinguish between the HCC cells and hepatocytes.

**Fig. 2:**
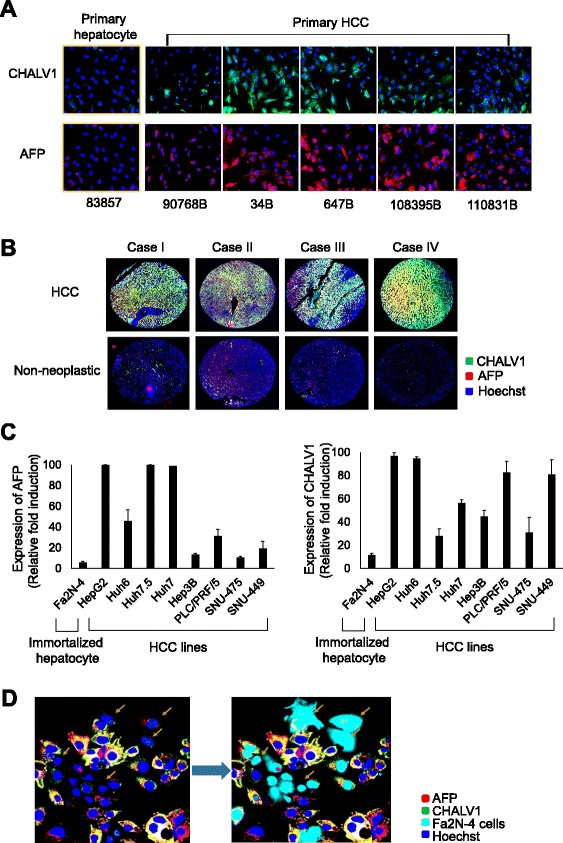
CHALV1 and AFP are cancer-specific markers for HCC cells. **a** Immunocytochemistry images of CHALV1 and AFP in primary hepatocytes (83857) and primary HCC cells, which were dissociated from liver resection specimens of liver cancer patients. **b** Representative immunohistochemistry images of CHALV1 and AFP in liver cancer tissues and surrounding normal tissues. **c** Expression of CHALV1 and AFP in hepatocyte (Fa2N-4), hepatoblastoma (Huh6), and HCC cell lines. **d** Fluorescence images of AFP and CHALV1 in a mixed culture system with CellLight® Nucleus-GFP-labeled Fa2N-4 cells (*sky blue*) and Huh7 cells. All images and graphs were analyzed using the Operetta® High Content Screening System. Images in the same panel were obtained with same magnification

In this study, we selected two distinctive markers of HCC, hepatocellular carcinoma monoclonal antibody (CHALV1) and α-fetoprotein (AFP). CHALV1 was raised against liver carcinoma cells of human origin and AFP is routinely used for liver cancer diagnosis. To confirm physiological relevancy, we detected CHALV1 and AFP in primary hepatocytes (83857) and primary hepatocellular carcinoma tumors (90768B, 34B, 647B, 108395B, and 110831B) which were isolated from liver resection specimens of liver cancer patients. Expression of CHALV1 and AFP was markedly higher in primary hepatocellular carcinomas than in primary hepatocytes (83857) (Fig. 2a). Immunohistochemical analysis revealed that HCC tissues had an abundance of CHALV1^+^/AFP^+^ cells relative to the surrounding normal tissues (Fig. 2b, Additional file 1: Figure S1). We observed that the expression of CHALV1 or AFP was also relatively higher in HCC lines than in Fa2N-4 cells, an immortalized human hepatocyte line (Fig. 2c, Additional file 1: Figure S2). Next, we sought to determine whether CHALV1 or AFP could distinguish between HCC cells and hepatocytes. To this end, we created a co-culture system using Huh7 HCC cells and Fa2N-4 cells that were labeled with CellLight® Nucleus-GFP (Additional file 1: Figure S3). Thus, the difference between HCC cells and hepatocytes was easily discernible in this co-culture culture system.

In Fig. 2d, the yellow arrows indicate cells with low expression of the HCC markers (CHALV1^−^/AFP^−^), which might be assumed to be the immortalized normal hepatocytes. Indeed, there was an overlap between the indicated cells and CellLight® Nucleus-GFP labeled-Fa2N-4 cells. Therefore, we concluded that CHALV1 and AFP are appropriate markers to distinguish between HCC cells and hepatocytes for the screening of HCC-specific compounds.

### Identification of compounds targeting HCC cells versus normal hepatocytes in a mixed population

We designed a co-culture-based drug screening system, using Fa2N-4 (immortalized hepatocytes) and Huh7 cells (HCC cells) to identify compounds that selectively target HCC cells. Because this screening system was created by mixing of two kinds of cell lines which have different properties, we tried to find appropriate culture conditions that maintained the individuality of both cell types. Fa2N-4 cells are generally cultured in serum-free supporting culture medium (SF- medium). When Fa2N-4 cells were maintained in DMEM supplemented with heat-inactivated 10 % FBS, we observed a change in Fa2N-4 morphology. Despite the change in culture medium, Huh7 cells maintained their morphology (Additional file 1: Figure S4A). Expression of AFP in Huh7 cells was not changed when medium was changed from DMEM supplemented with heat-inactivated 10 % FBS to SF- medium (Additional file 1: Figure S4B).

Next, we estimated the doubling time of Huh7 cells and Fa2N-4 cells to decide the proper mixing ratio for the co-culture system. The doubling time of Huh7 cells was 23.8 h and the doubling time of Fa2N-4 cells as 43.5 h (Additional file 1: Figure S4C). Based on this, the cells were premixed at a ratio of Fa2N-4: Huh7 cells of 65:35 and plated in a randomly mixed state. Additionally, we confirmed that swapping of medium did not have an effect on the cell doubling time in both cell lines (data not shown). Therefore, we used SF- medium for the co-culture screening system.

**Fig. 3:**
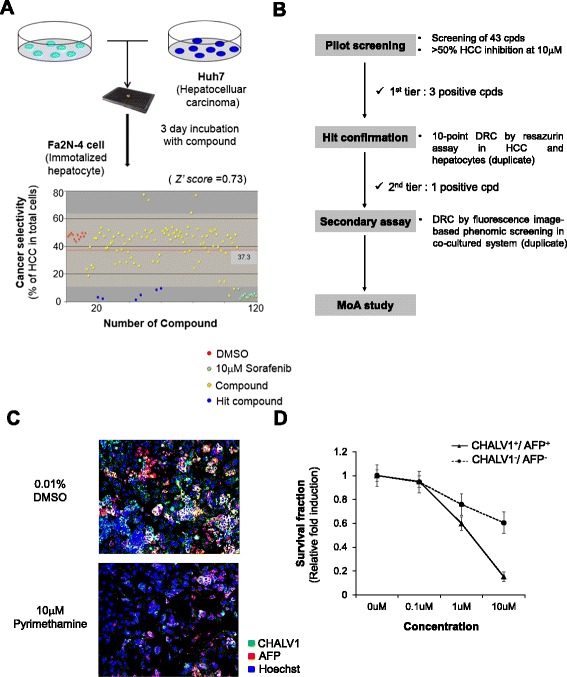
Identification of compounds that are selective for HCC. **a** Schematic of screening of HCC selective compounds in a mixed culture system (Fa2N-4 and Huh7 cells) and screening data. 0.01 % of DMSO was used as negative control (*red circle*), 10 μM of sorafenib was used as positive control (*green circle*) and hit compounds (*Blue circle*). **b** Schematic of the drug discovery process, from pilot screening to the mechanism of action study. **c** Immunofluorescence images of the mixed culture system after treatment with DMSO and 10 μM of pyrimethamine. Images were obtained with same magnification. **d** Viability of Huh7 (CHALV1^+^/AFP^+^) and Fa2N-4 (CHALV1^−^/AFP^−^) cells in the mixed culture system using image-based analysis. Experiments were performed in triplicate. Error bars indicate standard deviation

Based on these results, we set up well-defined mixed HCC cell populations using Huh7 cells and Fa2N-4 cells in 384 well plates for image-based phenotypic screening. To confirm whether developed co-culture system detect selective effect between anticancer effect and drug induced hepatotoxicity, we configured the small screening library including largely two group. Class I includes approved anticancer drugs and cytotoxic compounds whereas Class II includes withdrawn or not marked due to hepatotoxicity and marketed with hepatotoxicity warnings in their labels. The mixed HCC cell populations were incubated for 3 days with 43 compounds (Fig. 3a, Additional file 1: Figure S5). After treatment, the cells were labeled with antibodies against CHALV1 and AFP and images were acquired. Small molecular weight compounds which only affected HCC cells were selected by the Harmony 3.5.1® high content imaging and analysis software. We determined the percentage of Huh7 cells to measure anticancer activity (as cancer selectivity) and the percentage of Fa2N-4 to detect hepatotoxicity based on the meaning Z’ score = 0.73 (Fig. 3a). Through the pilot screening, we found HCC-specific drugs (methotrexate, pyrimethamine, and aminopterin, all anti-folate drugs) that significantly induced cell death in HCC (CHALV1^+^/AFP^+^) cells while minimizing the damage to hepatocytes (CHALV1^−^/AFP^−^) (Fig. 3b, Table 1). To confirm the HCC selectivity of anti-folate drugs, we estimated the growth inhibitory effects by a resazurin assay in an individual culture system. Among the drugs tested, pyrimethamine displayed the greatest cancer-specific cytotoxicity, with approximately 8-fold higher cytotoxicity in Huh7 cells than in Fa2N-4 cells (Table 1). When pyrimethamine was applied to co-cultures of HCC cells and hepatocytes, the reduction in CHALV1^+^/AFP ^+^ cells was significantly greater than the reduction in CHALV1^−^/AFP^−^ cells (Fig. 3c). A dose response curve also showed that pyrimethamine increased cell death in HCC cells compared to normal hepatocytes. These results showed that pyrimethamine selectively suppressed the HCC population in a co-culture system of HCC cells with hepatocytes.

**Table 1:** Half maximal inhibitory concentrations (IC_50_) of anti-folates in Fa2N-4 and Huh7 cell lines

		IC_50_(μM)		* IC_50_ER
	Fa2N-4		Huh7	
MTX	0.01518		0.004074	3.726068
Pyrimethamine	23.43		2.764	8.476845
Aminopterin	0.001688		0.0005194	3.249904

*IC_50_ER : IC_50_ with enhancement ratio (Fa2N-4/Huh7)

### Pyrimethamine induces growth inhibition by DNA damage and S-phase cell cycle arrest without ROS accumulation and mitochondrial dysfunction

**Fig. 4:**
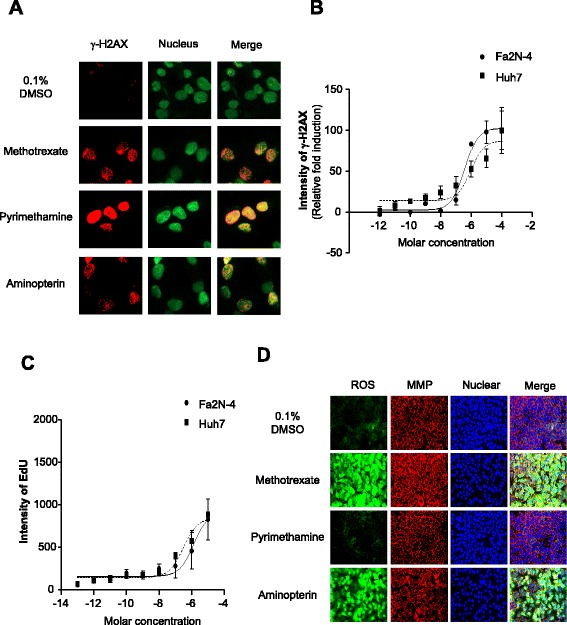
Pyrimethamine induces DNA damage and S-phase arrest, but does not affect ROS accumulation and mitochondrial dysfunction. **a** Immunocytochemistry images of γ-H2AX in Huh7 cells after 24 h incubation with the anti-folate drugs at a concentration of 10 μM. **b** Intensity of γ-H2AX in the Fa2N-4 and Huh7 cell lines after treatment with pyrimethamine at the indicated concentrations. Experiments were performed in triplicate. Error bars indicate standard deviation. **c** Analysis of proliferation activity in Huh7 and Fa2N-4 cells using EdU staining after 24 h incubation with pyrimethamine at the indicated concentrations. **d** ROS and mitochondrial membrane potential (MMP) were measured after 24 h incubation with anti-folate drugs. ROS and MMP were detected by staining with CM-H_2_DCFDA (*green*) and tetramethylrhodamine, methyl ester (TMRM; *red*). All images and analyses were examined using the High Content Screening System. Images in the same panel were obtained with same magnification

To determine the mechanism of pyrimethamine-induced tumor-specificity in HCC, we applied various cell-based assay models in vitro to the image-based phenotypic analysis system in order to visualize the pyrimethamine-induced phenotypic changes and cytotoxic effects. First, we compared the level of γ-H2AX in Huh7 and Fa2N-4 cells, because pyrimethamine can inhibit DNA synthesis by blocking the enzymatic activity of dihydrofolate reductase. Among the three anti-folate drugs, pyrimethamine displayed the highest intensity of γ-H2AX staining, because γ-H2AX did not appear as foci, but rather as a diffuse pattern (Fig. 4a). However, pyrimethamine induced similar amounts of H2AX phosphorylation in Huh7 and Fa2N-4 cells (Fig. 4b).

To investigate the mechanism of cell growth inhibition induced by pyrimethamine, pyrimethamine-treated Huh7 and Fa2N-4 cells were subjected to an EdU proliferation assay. The EdU proliferation assay revealed that pyrimethamine also similarly increased the number of EdU-positive cells in both cell lines (Fig. 4c). Thus, pyrimethamine did not have a differential effect on the level of DNA damage and S-phase cell cycle arrest between HCC cells and hepatocytes.

To further investigate the mechanism of HCC-specific cytotoxicity triggered by pyrimethamine, we analyzed phenotypic changes in cell organelles, because damaged organelles can trigger tumor cell death. Oxidative stress, mitochondrial damage, and lysosome distribution were measured using the image-based phenotypic analysis system in pyrimethamine-treated Huh7 and Fa2N-4 cells. Reactive oxygen species (ROS) are important signaling molecules in normal conditions, but their accumulation in pathological conditions leads to oxidative stress. Because ROS can provide important clues about the physiological status of the cell, its production was assessed. Compared with the cells treated with pyrimethamine, other anti-folate drugs, including methotrexate and aminopterin, induced a significantly high production of ROS in a dose-dependent manner, which was detected by the permeable and redox-sensitive dye CM-H_2_DCFDA after 24 h of anti-folate treatment. However, pyrimethamine did not enhance ROS accumulation at wide range of concentrations in both cell lines (Fig. 4d, Additional file 1: Figure S6A).

Next, we assessed mitochondrial function, which is a key factor in the regulation of apoptotic cell death. MMP (ΔΨm) is critical for maintaining the physiological function of the respiratory chain to generate ATP. A significant loss of ΔΨm renders cells depleted of energy, leading to subsequent death. We examined the effect of pyrimethamine on the ΔΨm by TMRM (tetramethylrhodamine, methyl ester) staining. However, as shown in Fig. 4d and Additional file 1: Figure S6B, the ΔΨm was not changed by pyrimethamine. These results showed that pyrimethamine-mediated HCC-specific cytotoxicity has no connection with growth inhibition by DNA damage, ROS accumulation, or mitochondrial dysfunction.

### Pyrimethamine induces an increase in the size and number of lysosomes and subsequently induces strong activation of cathepsin B in Huh7 but not Fa2N-4 cells

**Fig. 5:**
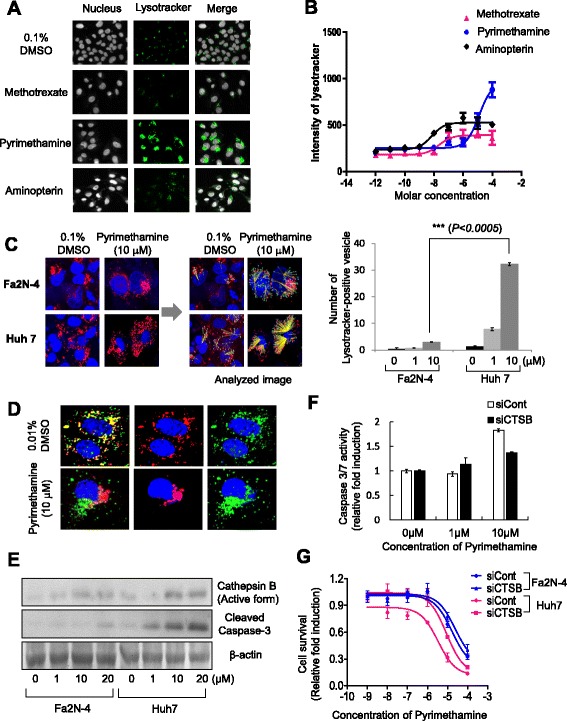
Pyrimethamine induces lysosomal modification and release of cathepsin B in HCC cells. **a** Fluorescence images of lysosomes (LysoTracker® Green DND-26; *green*) and **b** intensity of LysoTracker in the Huh7 cell line after anti-folate drug treatment (x-axis indicates molar concentration). **c** Lysosome staining images (LysoTracker® Red DND-99, red) of the Fa2N-4 and Huh7 cell lines after pyrimethamine treatment (left; 48 h, 10 μM) and image analysis of LysoTracker Red-positive vesicle number (*right*). Graph represents LysoTracker-positive vesicles in the Fa2N-4 and Huh7 cell lines after pyrimethamine treatment (*bottom*). **d** Translocation of cathepsin B was detected in the Huh7 cell line after pyrimethamine treatment (48 h, 10 μM) and **e** expression of active cathepsin B and active caspase-3 detected by Western blot analysis after incubation with pyrimethamine at the indicated concentration. **f** The level of active caspase 3/7 in Huh7-siCont and Huh7-siCTSB cells after pyrimethamine treatment at 1, 10 μM concentration. **g** Drug response curves of Huh7-siCont, Huh7-siCTSB, Fa2N-4-siCont, and Fa2N-4-siCTSB cells after treatment of pyrimethamine at ten point concentrations (from 1 pM to 100 μM). All images and analyses were examined using the High Content Screening System. Experiments were performed in triplicate. Error bars indicate standard deviation. Images in the same panel were obtained with same magnification

Next, we detected morphological changes in lysosomes by LysoTracker, which labels acidic compartments in live cells. The untreated cells displayed numerous small LysoTracker-positive vesicles. Other anti-folate-treated cells showed few differences in the LysoTracker signal compared with the DMSO control. On the other hand, pyrimethamine treatment induced an enhancement in LysoTracker-labeled vesicle size, number, and LysoTracker intensity (Fig. 5a, b). Thus, we hypothesized that pyrimethamine induces apoptosis by causing lysosomal dysfunction in addition to the inhibition of DNA synthesis in HCC. Tumor cells have been reported to have larger lysosomes, which makes more susceptible to breakage. To determine whether the HCC-specific cytotoxicity of pyrimethamine was related to the number of lysosome vesicles, we compared the number of LysoTracker-positive vesicles in Huh7 and Fa2N-4 cells after pyrimethamine treatment. Interestingly, pyrimethamine more significantly increased the size and number of LysoTracker-positive vesicles in Huh7 cells than in Fa2N-4 cells (Fig. 5c). Recently, apoptotic stimuli have been shown to trigger lysosomal membrane permeability, leading to the release of cathepsins that can activate death signaling pathways in the cytosol. Specifically, the release of cathepsin B induces the activation of caspase-3 and −9, thereby leading to apoptosis. Based on this background, we analyzed cathepsin B localization in Huh7 cells, before and after the pyrimethamine treatment. The untreated cells displayed a yellow punctate pattern, because cathepsin B was localized to the lysosome vesicular compartment. However, pyrimethamine triggered the release of cathepsin B from the lysosome to cytosol (Fig. 5d). Next, we detected the active form of cathepsin B and the cleavage of caspase-3 in Fa2N-4 and Huh7 cells. Indeed, pyrimethamine treatment induced cathepsin B activation and caspase-3 cleavage in Huh7 cells to a greater extent than in Fa2N-4 cells (Fig. 5e).

To confirm whether pyrimethamine-mediated cathepsin B activation is responsible for the apoptosis in HCC, cathepsin B siRNA (siCTSB) was transfected to Fa2N-4 and Huh7 cells. Cathepsin B depletion significantly decreased the activity of caspase 3/7 by treatment of pyrimethamine in Huh7 cells (Fig. 5f). Additionally, the IC_50_ value of pyrimethamine was increased by cathepsin B depletion (from 3.4 to 8.8 μM) in Huh7, but not in Fa2N-4 (Fig. 5g). From these results, it appears that the tumor-specific cytotoxicity of pyrimethamine is mediated by the enhancement of cathepsin B activity in HCC cells.

### Pyrimethamine exhibits an anti-tumor effect on Huh7 spheroids without toxicity to Fa2N-4 spheroids

**Fig. 6:**
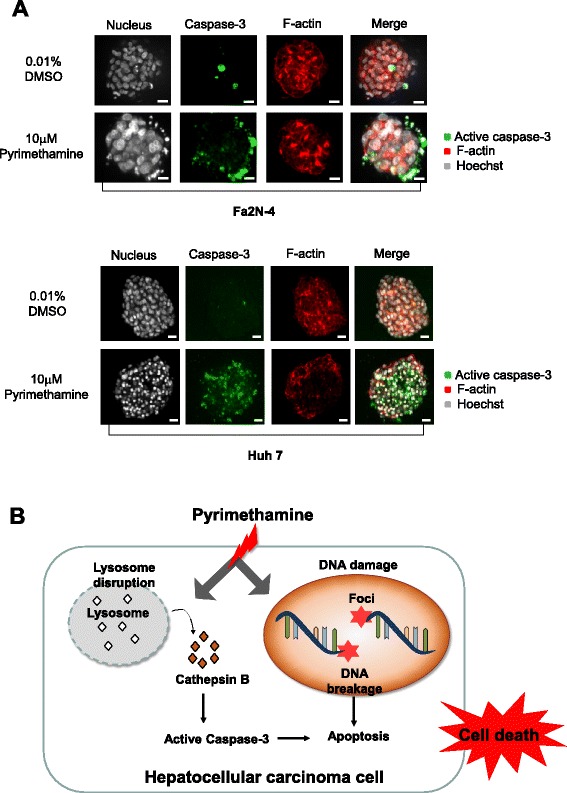
Pyrimethamine has anticancer effects against Huh7 cells in a 3D culture system. Fluorescence images of cleaved caspase-3, which is an apoptosis marker, and F-actin, which detects bile canaliculus structures, in (**a**) Fa2N-4 and (**b**) Huh7 spheroids after pyrimethamine treatment (10 μM, 7 days). **c** Schematic illustrations of mechanisms by which pyrimethamine induces HCC-specific toxicity based on the results. Scale bar = 200 μm

In recent years, a paradigm shift from two-dimensional (2D) to 3D cell culture techniques has occurred, because 2D cell-based models fail to predict in vivo efficacy, contributing to the low success rate of translating an investigational new drug to clinical approval. In order to overcome the shortcomings of 2D culture during drug discovery, we used a 3D culture system, which is the appropriate culture model to sustain liver-specific functions that are more representative of in vivo models. Huh7 spheroids and Fa2N-4 spheroids were exposed to pyrimethamine for 7 days. For the analysis of cytotoxicity in both spheroids, we extracted the middle image from 50 of the 3D stack images. Typically, only a few cells became cleaved caspase-3-positive in untreated spheroids during the course of their maturation, whereas anti-folate drug-treated spheroids were composed mostly of cells with activated caspase-3 (Fig. 6a, Additional file 1: Figure S7). Of note, pyrimethamine induced a strong increase in caspase-3 in Huh7 spheroids compared with Fa2N-4 spheroids (Fig. 6a). Additionally, we investigated the effect of pyrimethamine on the F-actin pattern between Huh7 and Fa2N-4 spheroids, because bile canaliculi, which have an important role in the maintenance of liver function, contain many F-actin microfilaments. Pyrimethamine destroyed bile canaliculi-like architecture in Huh7 spheroids. However, Fa2N-4 spheroids maintained their pattern of F-actin staining after treatment with pyrimethamine (Fig. 6a). These results showed that pyrimethamine induced greater cytotoxicity on HCC cells than on hepatocytes in 3D culture.

## Discussion

HCC is the fourth most common form of cancer in the world, with an incidence of 2.1 in 100,000 in North America and 80 in 100,000 in China [16]. Because only 10–20 % of liver tumors can be removed surgically, the prognosis for this disease is very poor. The major causes of death are chronic infection by hepatitis B and C, and the metastases of tumors from elsewhere in the body [17]. For most liver cancer patients, treatment options are limited to palliative care, because liver cirrhosis causes the loss of main liver functions including detoxification, protein synthesis, and the production of biochemicals necessary for digestion.

Moreover, most HCCs are resistant to conventional chemotherapeutic agents. Cytotoxic drugs such as doxorubicin or cisplatin have no efficacy in the treatment of HCC. Recently, sorafenib, which targets the Raf kinase, VEGFR, and PDGFR signaling pathways, was approved for the treatment of patients with advanced HCC [2, 18]. Generally, sorafenib treatment costs US $5,400 per month, but it only extends lifespan by an average of 2.8 months with various side effects. Therefore, the search for new anticancer drugs for HCC is critical.

Previously, we developed target-based and phenotypic screening approaches for drug discovery and basic research into disease mechanisms [19, 20]. The phenotypic screening approach was the most successful approach for first-in-class drugs. Such screens can lead to the identification of novel targets, novel compound mechanisms of action, and new pathways of therapeutic value [21].

In present study, we found visual signatures of HCC cells and hepatocytes, and validated assays to screen for anti-HCC compounds. We obtained hepatocytes labeled with CellLight® Nucleus-GFP and mixed them with HCC cells. These mixtures were used as a training set for image-based supervised learning to develop the imaging analysis algorithms to detect HCC cells in a background of non-cancerous cells (Fig. 2d). Because our ultimate goal was to define visual parameters that could be used in the absence of a fluorescent marker, we subsequently identified two distinctive markers of HCC, CHALV1 and AFP, which were detected in HCC cell lines and primary HCC tumors (Fig. 2). CHALV1 and AFP had not yet been used for HCC drug screening, but we anticipate that they will become valuable biomarkers for predicting the risk of HCC in the foreseeable future. We developed heterotypic, mixed HCC cell populations using immortalized hepatocytes and HCC cells for HCC drug discovery (Fig. 3a). Cells were premixed at a Fa2N4:Huh7 cell ratio of 65:35, plated in a randomly mixed state, and incubated with cytotoxic compounds for 3 days.

Our HCC co-culture system represents a powerful method for detecting the selectivity index between anticancer activity and drug-induced liver toxicity. Moreover, this system can simply be used to elucidate heterotypic intercellular interactions during physiological and pathological processes. For the pilot screening, we treated with compounds from a small chemical library, which included class I compounds, consisting of clinically approved anticancer drugs and cytotoxic compounds, and class II compounds, consisting of hepatotoxic compounds (Additional file 1: Figure S5).

Through the pilot screening, we identified cancer-specific anti-folate drugs such as methotrexate, pyrimethamine, and aminopterin. Over the past few years, several studies have been done on the anticancer effects of pyrimethamine [11, 13, 22, 23]. However, the cancer-specific effects of pyrimethamine have never been studied in HCC. Here, pyrimethamine had the highest cancer selectivity among anti-folate drugs; however, it was only effective at high concentrations (Table 1).

We determined morphological changes of cell organelles, because organelle damage responses can be used to trigger tumor cell death. Oxidative stress, mitochondrial damage, lysosome distribution, and cell number were measured by the phenotypic analysis system in HCC.

Studies have shown that anti-folate drugs are involved in the de novo biosynthesis of purines and pyrimidines, thereby inducing an imbalance in the nucleotide pool, which leads to consequent DNA damage and S-phase cell cycle arrest. However, the level of DNA damage and S-phase cell cycle arrest was not significantly different between hepatocytes and HCC cells after pyrimethamine treatment (Fig. 4a, b, c). Moreover, because methotrexate induces ROS production [24, 25], we investigated the possibility that pyrimethamine was involved in ROS production. The accumulation of ROS was observed in cells treated with methotrexate and aminopterin. However, pyrimethamine treatment did not induce ROS production (Fig. 4d, Additional file 1: Figure S6A).

Lysosomes are promising potential drug targets and mediators of apoptosis signaling [26, 27]. Tumor cell lysosomes contain increased levels of cathepsins, and the release of these enzymes into the cytosol may result in apoptosis or necrosis [28, 29]. In Fig. 5a and c, we ascertained that pyrimethamine mediated lysosomal modification, which included alterations in the size and number of lysosomes. Of note, pyrimethamine mediated lysosomal modification, the release of cathepsin B from lysosomes to the cytoplasm, and the activation of caspase-3 to a greater extent in HCC cells than in hepatocytes (Fig. 5d).

To overcome the drawbacks of 2D culture, we built a cellular assay using 3D cell culture systems, which are more predictive of the in vivo and clinical responses. Liver cells in 3D culture, which remain viable and retain differentiated functions for a longer period of time, have a potential use for toxicity testing [30]. In the liver, hepatocytes polarize epithelial cells whose plasma membranes consist of two morphologically and functionally distinct domains: basolateral and bile canalicular membranes. Specifically, bile canaliculi (BC) play an important role in hepatic bile secretion. BC contraction is one of the functions of the liver at the tissue level, because it is needed to have communication and integration among neighboring hepatocytes [31, 32]. Therefore, we detected hepatic F-actin to estimate the effects of drugs on BC alteration and activated caspase-3, which is a hallmark of apoptosis in 3D spheroids. As shown in Fig. 6a, pyrimethamine also strongly induced cancer-specific cytotoxicity in 3D cell culture systems. A plausible mechanism for pyrimethamine-induced cancer selectivity in HCC is shown in Fig. 6b, which is mediated by lysosomal modification (increases in cathepsin B and cleaved caspase-3) and DNA damage. These results suggest that pyrimethamine may have a novel anticancer therapeutic use in liver cancer therapy.

## Conclusions

In this paper, we have developed an in vitro co-culture system of HCC cells with hepatocytes to recapitulate tumor heterogeneity and have applied this co-culture system to an image-based phenotypic drug screening platform for the development of HCC therapeutics. We have also identified HCC-specific markers, CHALV and AFP, to distinguish between cell types in this platform. Based on the image-based phenotypic analysis, we promptly estimated anticancer activity and hepatotoxicity and elucidated functional roles of pyrimethamine during the apoptosis process in HCC. This in vitro screening platform has the potential to be widely adopted in drug discovery research due to its easy handling and efficiency.

## Additional file

Additional file 1: Figure S1. Immunocytochemistry images of CHALV1 and AFP in HCC cell lines. HCC cell lines were stained with CHALV1 (green) and AFP (red) antibody, and Fa2N-4 cells were used as negative control. **Figure S2** Immunohistochemistry images of CHALV1 and AFP in various liver cancer tissues and their surrounding normal tissue. Slide was immunostained with CHALV1 and AFP in 32 cases Image acquisition was performed using high content screening system. **Figure S3** Distinguishable staining of Fa2N-4 for more accurate analysis. Fluorescence images of Fa2N-4 labeled with CellLight® Nucleus-GFP (sky blue) and Hoechst33342 (deep blue). **Figure S4** Examination of cell phenotype through swapping of medium. (A) Bright field images of Fa2N-4 and Huh7 cells were tested in serum free supporting Fa2N-4 cells (SF) culture medium and DMEM medium. (B) Western blot analysis of AFP expression of Fa2N-4 and Huh7 cell line, which were cultivated in SF and DMEM medium. (C) Doubling time of Fa2N-4 and Huh7 cell lines. **Figure S5** List of 43 compounds used for image-based phenotypic screening. Class I are list of clinical approved anticancer drugs and cytotoxic compounds and Class II are list of hepatotoxic compounds. **Figure S6** Level of ROS accumulation and mitochondrial membrane potential in Fa2N-4 and Huh7 cells with anti-folate drug. Examination of (A) ROS accumulation and (B) mitochondrial membrane potential were performed after 24 h.incubation with pyrimethamine. Based on the image, value was analyzed by Operetta. Experiments were performed in triplicate and error bars indicate standard deviation. (PPTX 16829 kb)

## Abbreviations

2D: Two dimensional
3D: Three dimensional
AFP: Alpha-fetoprotein
BC: bile canaliculi
BS solution: Bovine serum solution
CM-H_2_DCFDA: 5-(and-6)-chloromethyl-2′,7’-dichlorodihydrofluorescein diacetate, acetyl ester
DHFR: Dihydrofolate reductase
DRC: Dose response curve
EdU: 5-ethynyl-2’-deoxyuridine
FBS: Fetal bovine serum
GFP: Green fluorescent protein
HCC: Hepatocellular carcinoma
HCS: High content screening
LMP: Lysosomal membrane permeability
LYSOTRACKER: Lysotracker red
MMP (ΔΨm): Mitochondria membrane potential
MoA: Mode of action
PBS: Phosphate buffered saline
ROS: Reactive oxygen species
SF: Serum free supporting Fa2N-4 cells
TMRM: Tetramethylrhodamine, methyl ester
