# Supplementary material for: Hepatocellular carcinoma-targeted drug discovery through image-based phenotypic screening in co-cultures of HCC cells with hepatocytes
Source: BMC Cancer. 2016 Oct 18;16:810. doi: 10.1186/s12885-016-2816-x (PMC5069815; doi:10.1186/s12885-016-2816-x)
Supplement: Additional file 1: Figure S1. — Immunocytochemistry images of CHALV1 and AFP in HCC cell lines. HCC cell lines were stained with CHALV1 (green) and AFP (red) antibody, and Fa2N-4 cells were used as negative control. Figure S2 Immunohistochemistry images of CHALV1 and AFP in various liver cancer tissues and their surrounding normal tissue. Slide was immunostained with CHALV1 and AFP in 32 cases Image acquisition was performed using high content screening system. Figure S3 Distinguishable staining of Fa2N-4 for more accurate analysis. Fluorescence images of Fa2N-4 labeled with CellLight® Nucleus-GFP (sky blue) and Hoechst33342 (deep blue). Figure S4 Examination of cell phenotype through swapping of medium. (A) Bright field images of Fa2N-4 and Huh7 cells were tested in serum free supporting Fa2N-4 cells (SF) culture medium and DMEM medium. (B) Western blot analysis of AFP expression of Fa2N-4 and Huh7 cell line, which were cultivated in SF and DMEM medium. (C) Doubling time of Fa2N-4 and Huh7 cell lines. Figure S5 List of 43 compounds used for image-based phenotypic screening. Class I are list of clinical approved anticancer drugs and cytotoxic compounds and Class II are list of hepatotoxic compounds. Figure S6 Level of ROS accumulation and mitochondrial membrane potential in Fa2N-4 and Huh7 cells with anti-folate drug. Examination of (A) ROS accumulation and (B) mitochondrial membrane potential were performed after 24 h.incubation with pyrimethamine. Based on the image, value was analyzed by Operetta. Experiments were performed in triplicate and error bars indicate standard deviation. (PPTX 16829 kb) [file 12885_2016_2816_MOESM1_ESM.pptx]

## Slide 1
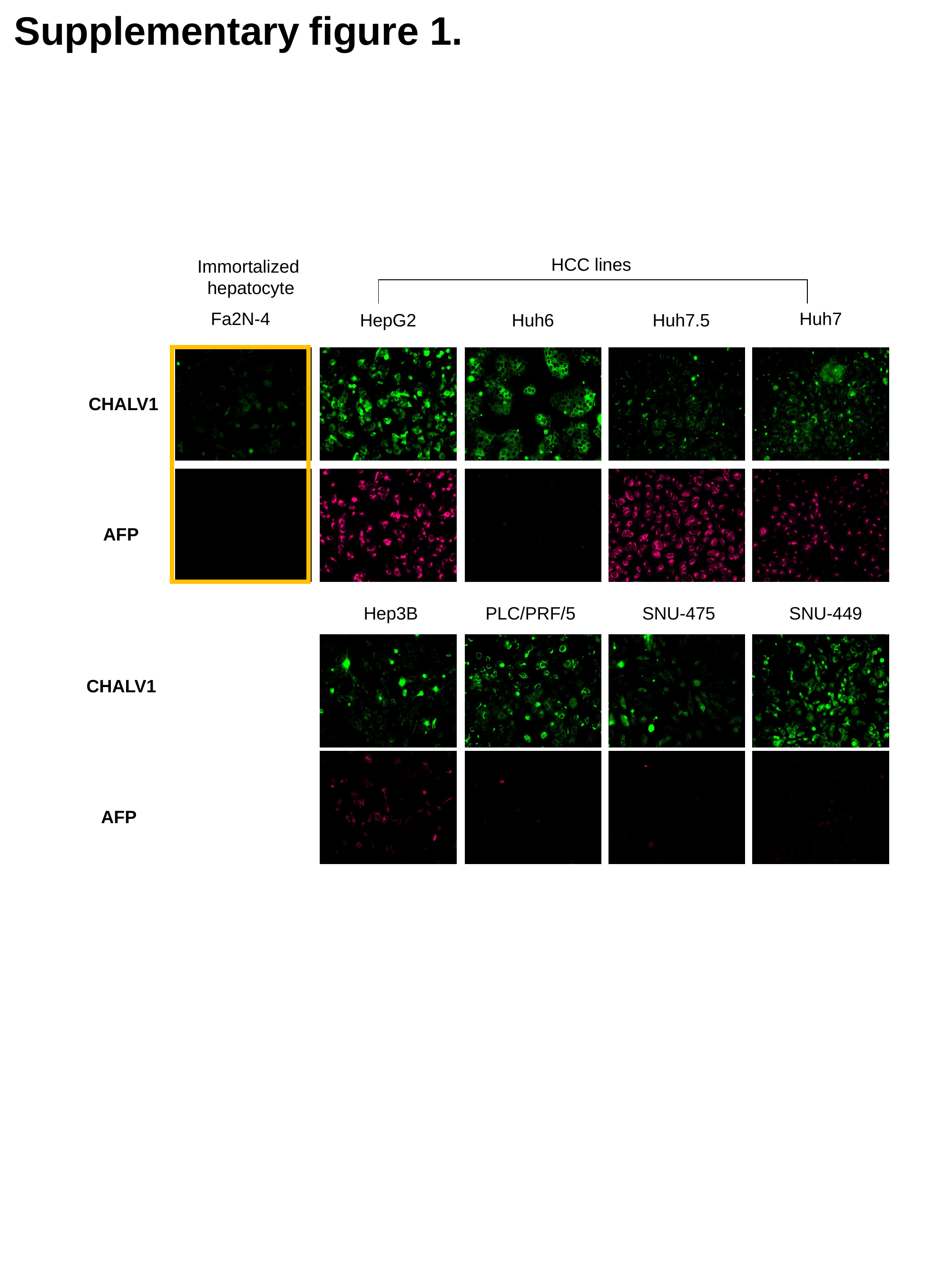

Supplementary figure 1.
 HCC lines
Immortalized
hepatocyte
Fa2N-4
Huh7
Huh7.5
HepG2
Huh6
CHALV1
AFP
Hep3B
PLC/PRF/5
SNU-475
SNU-449
CHALV1
AFP

## Slide 2
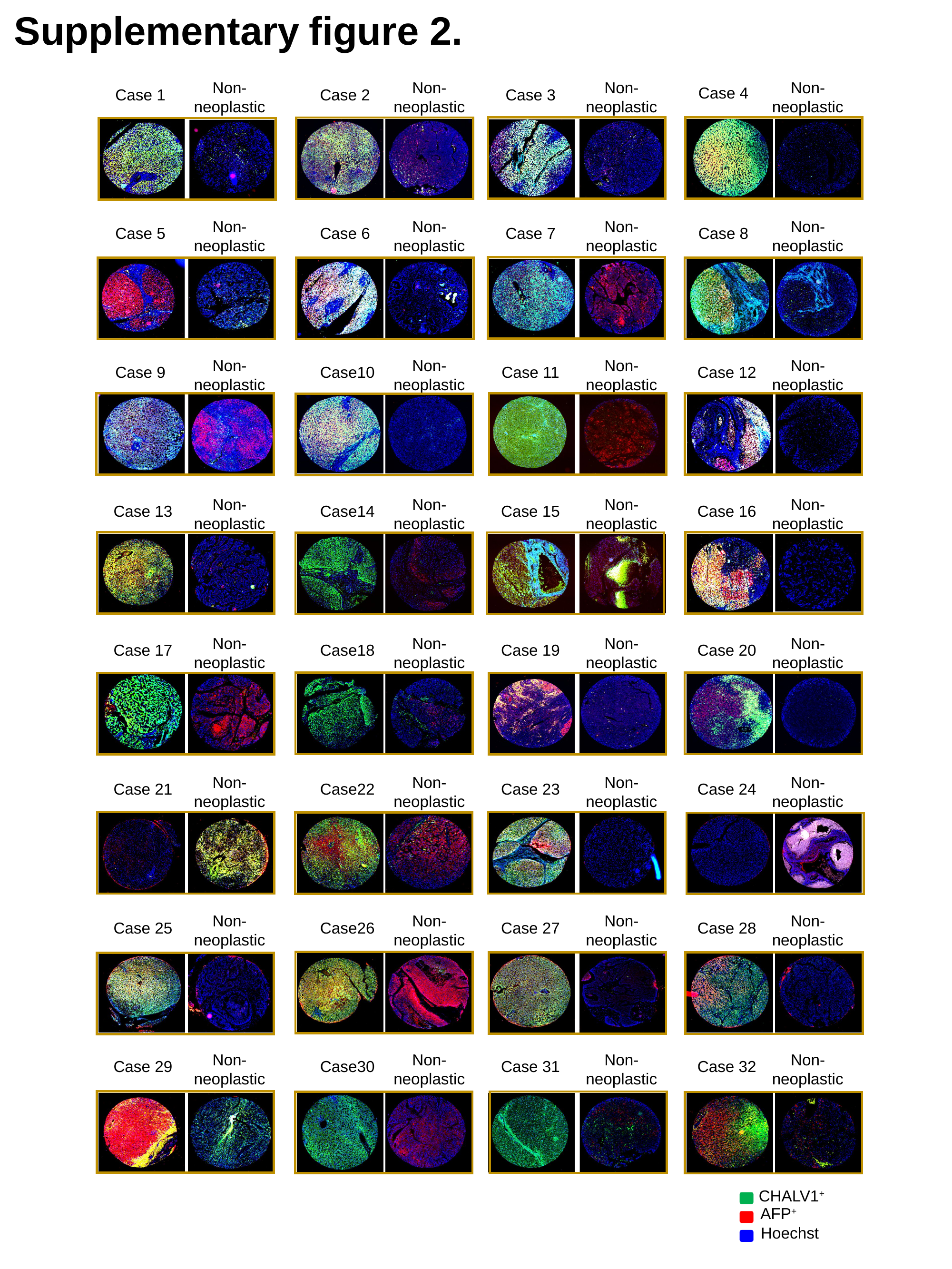

Supplementary figure 2.
Non-neoplastic
Non-neoplastic
Non-neoplastic
Non-neoplastic
Case 4
Case 1
Case 2
Case 3
Non-neoplastic
Non-neoplastic
Non-neoplastic
Non-neoplastic
Case 5
Case 6
Case 7
Case 8
Non-neoplastic
Non-neoplastic
Non-neoplastic
Non-neoplastic
Case 9
Case10
Case 11
Case 12
Non-neoplastic
Non-neoplastic
Non-neoplastic
Non-neoplastic
Case 13
Case14
Case 15
Case 16
Non-neoplastic
Non-neoplastic
Non-neoplastic
Non-neoplastic
Case 17
Case18
Case 19
Case 20
Non-neoplastic
Non-neoplastic
Non-neoplastic
Non-neoplastic
Case 21
Case22
Case 23
Case 24
Non-neoplastic
Non-neoplastic
Non-neoplastic
Non-neoplastic
Case 25
Case26
Case 27
Case 28
Non-neoplastic
Non-neoplastic
Non-neoplastic
Non-neoplastic
Case 29
Case30
Case 31
Case 32
CHALV1+
AFP+
Hoechst

## Slide 3
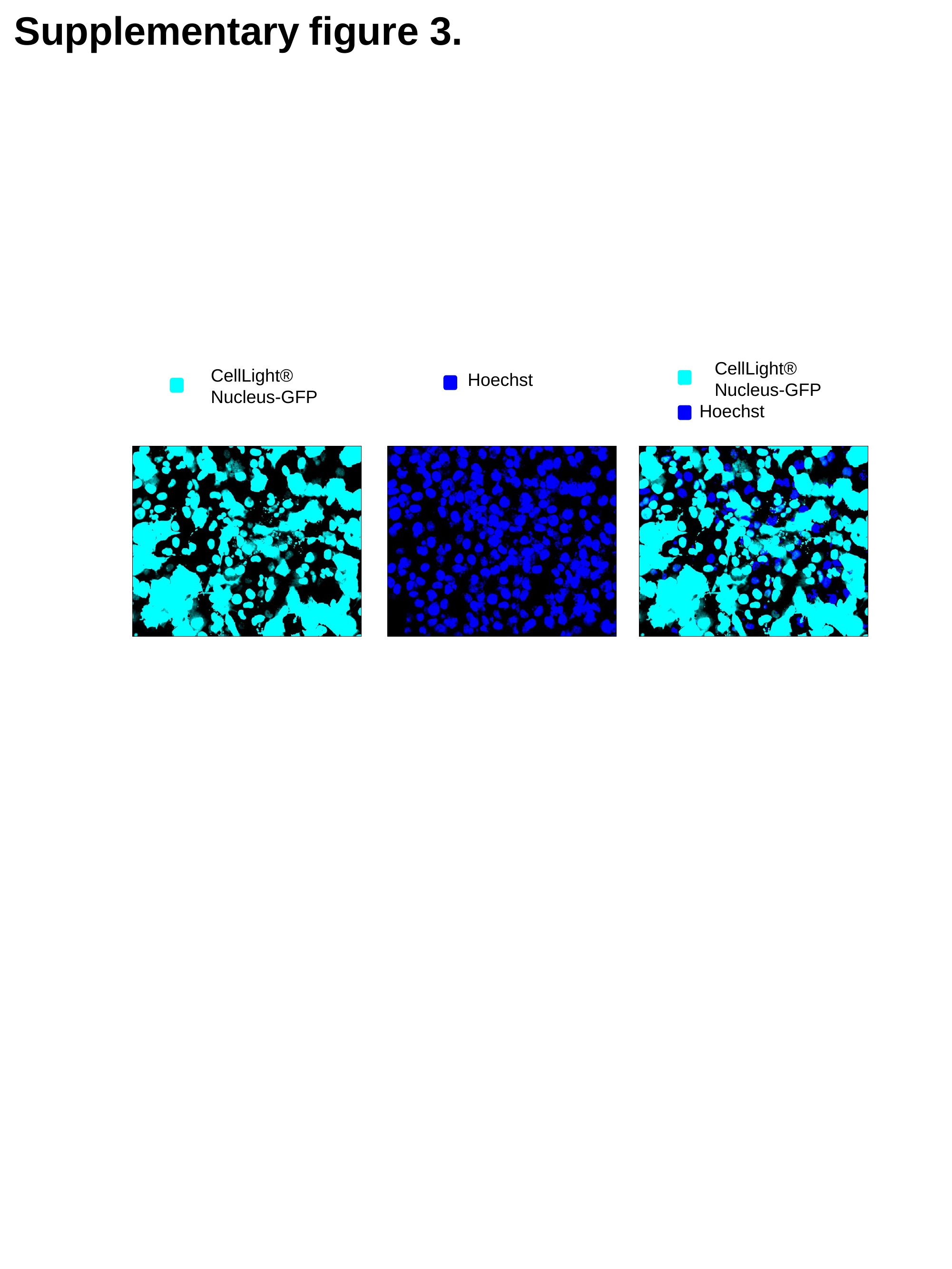

Supplementary figure 3.
CellLight®
Nucleus-GFP
CellLight®
Nucleus-GFP
Hoechst
Hoechst

## Slide 4
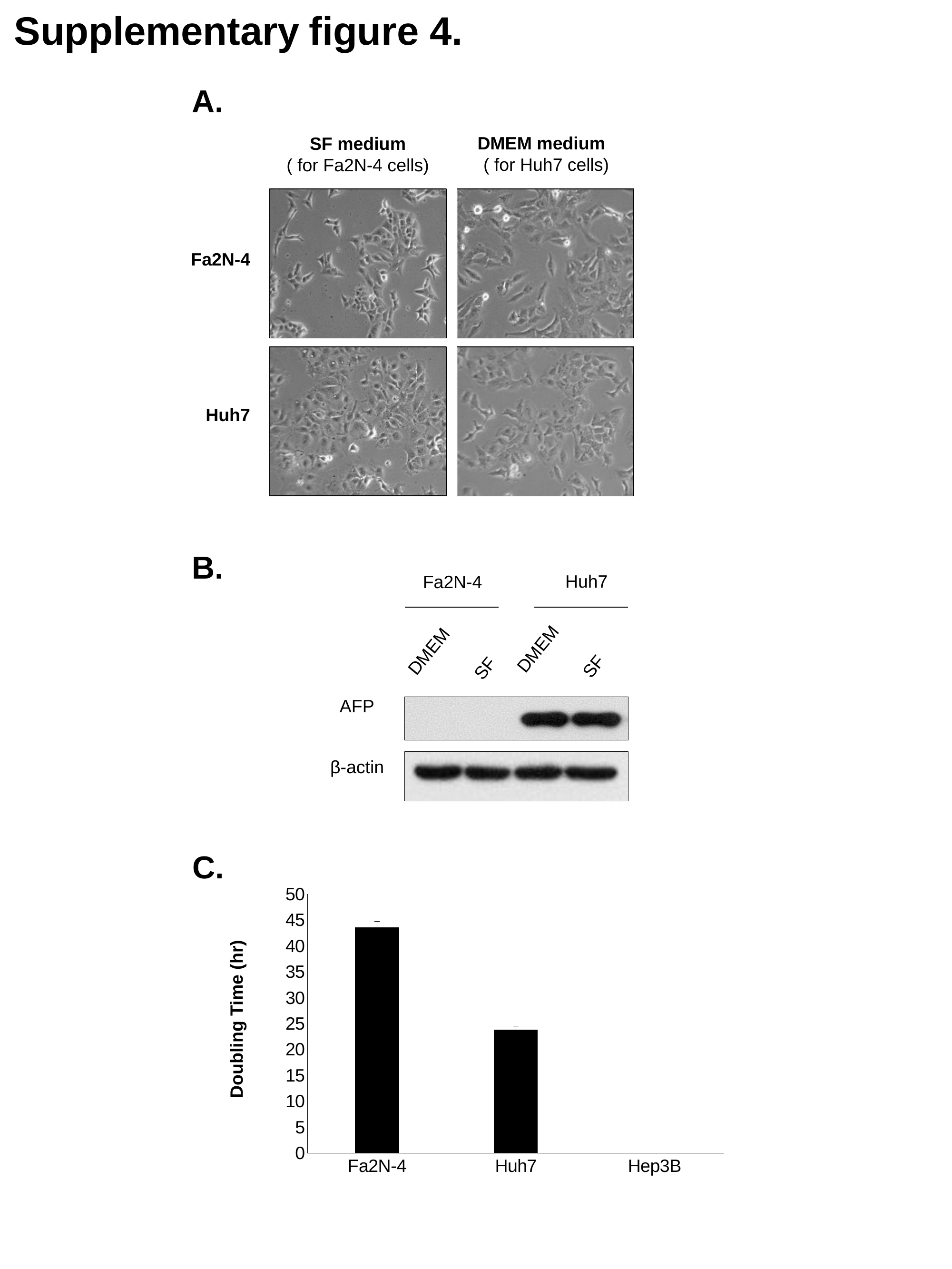

Supplementary figure 4.
A.
DMEM medium
( for Huh7 cells)
SF medium
( for Fa2N-4 cells)
 Fa2N-4
Huh7
B.
Huh7
Fa2N-4
DMEM
DMEM
SF
SF
AFP
β-actin
[unsupported chart]
C.
Doubling Time (hr)

## Slide 5
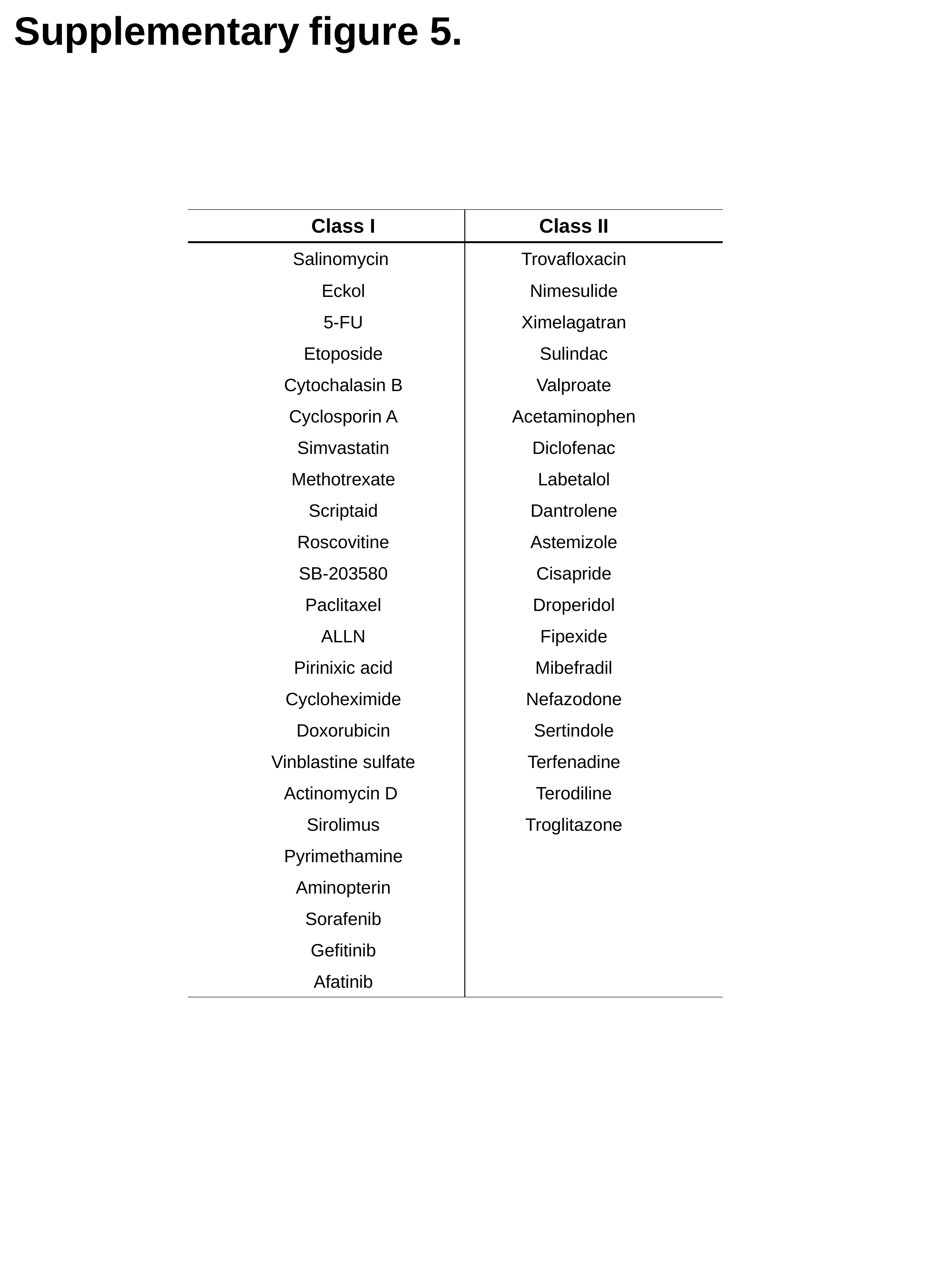

Supplementary figure 5.
| | Class I | Class II | |
| --- | --- | --- | --- |
| | Salinomycin | Trovafloxacin | |
| | Eckol | Nimesulide | |
| | 5-FU | Ximelagatran | |
| | Etoposide | Sulindac | |
| | Cytochalasin B | Valproate | |
| | Cyclosporin A | Acetaminophen | |
| | Simvastatin | Diclofenac | |
| | Methotrexate | Labetalol | |
| | Scriptaid | Dantrolene | |
| | Roscovitine | Astemizole | |
| | SB-203580 | Cisapride | |
| | Paclitaxel | Droperidol | |
| | ALLN | Fipexide | |
| | Pirinixic acid | Mibefradil | |
| | Cycloheximide | Nefazodone | |
| | Doxorubicin | Sertindole | |
| | Vinblastine sulfate | Terfenadine | |
| | Actinomycin D | Terodiline | |
| | Sirolimus | Troglitazone | |
| | Pyrimethamine | | |
| | Aminopterin | | |
| | Sorafenib | | |
| | Gefitinib | | |
| | Afatinib | | |

## Slide 6
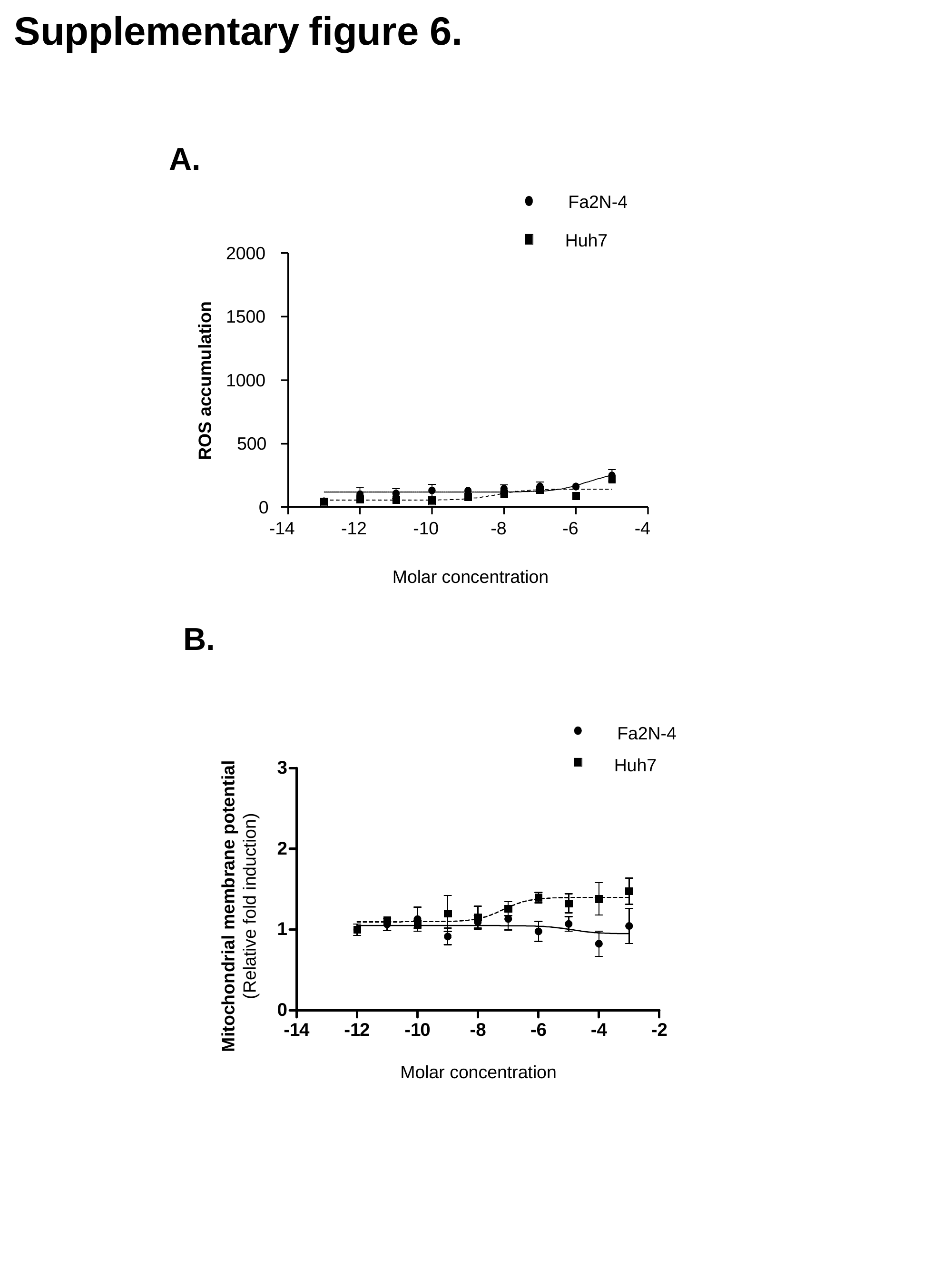

Supplementary figure 6.
A.
2000
1500
1000
500
0
-14
-12
-10
-8
-6
-4
Fa2N-4
Huh7
ROS accumulation
Molar concentration
Fa2N-4
Huh7
ROS accumulation
B.
Fa2N-4
Huh7
Mitochondrial membrane potential
(Relative fold induction)
Molar concentration

## Slide 7
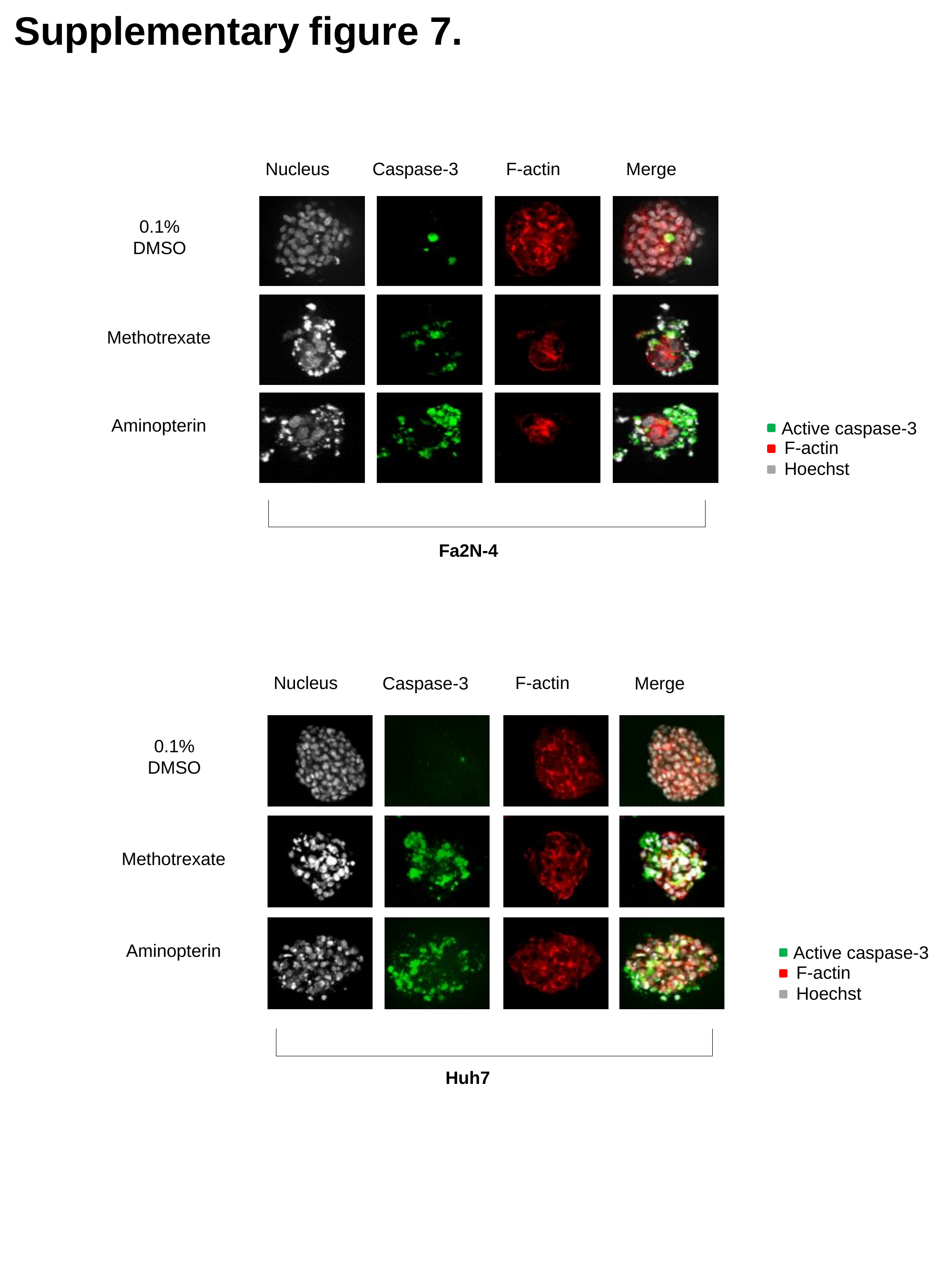

Supplementary figure 7.
Merge
Nucleus
Caspase-3
F-actin
0.1%
DMSO
Methotrexate
Aminopterin
Active caspase-3
F-actin
Hoechst
Fa2N-4
Nucleus
F-actin
Merge
Caspase-3
0.1%
DMSO
Methotrexate
Aminopterin
Huh7
Active caspase-3
F-actin
Hoechst
